# A nomogram for predicting the HER2 status in female patients with breast cancer in China: a nationwide, multicenter, 10-year epidemiological study

**DOI:** 10.1186/s13000-019-0806-4

**Published:** 2019-05-04

**Authors:** Huimin Zhang, Peiling Xie, Zhuoying Li, Rong Huang, Weiliang Feng, Yanan Kong, Feng Xu, Lin Zhao, Qingkun Song, Jing Li, Baoning Zhang, Jinhu Fan, Youlin Qiao, Xiaoming Xie, Shan Zheng, Jianjun He, Ke Wang

**Affiliations:** 1 Department of Breast Surgery, the First Affiliated Hospital of Xi’an Jiaotong University, 277 West Yanta Road, 710061 Xi’an, People’s Republic of China; 2 Department of Cancer Epidemiology, Cancer Institute & Hospital, Chinese Academy of Medical Sciences & Peking Union Medical College, Beijing, People’s Republic of China; 3 Department of Epidemiology, West China School of Public Health, Sichuan University, Chengdu, Sichuan People’s Republic of China; 4 Department of Breast Surgery, Zhejiang Cancer Hospital, Hangzhou, People’s Republic of China; 5 Department of Breast Oncology, Sun Yat-Sen University Cancer Center, Guangzhou, People’s Republic of China; 6 Department of Breast-thyroid Surgery, Xiangya Second Hospital, Central South University, Changsha, People’s Republic of China; 7 Department of Breast Surgery, Liaoning Cancer Hospital, Shenyang, People’s Republic of China; 8 Center of Breast Disease, Cancer Institute & Hospital, Chinese Academy of Medical Sciences & Peking Union Medical College, Beijing, People’s Republic of China; 9 Department of Pathology, Cancer Institute & Hospital, Chinese Academy of Medical Sciences & Peking Union Medical College, Beijing, People’s Republic of China

**Keywords:** Breast cancer, HER2 status, Nomogram, Prediction and validation

## Abstract

**Background:**

The concordance rate of human epidermal growth factor receptor 2 (HER2) status between core needle biopsy (CNB) and subsequent excisional biopsies of the same tumor varies from 81 to 96%, which may cause inappropriate neoadjuvant therapy that impair the potential benefit from HER2 targeted therapy for patients. This study aimed to establish a nomogram to predict the HER2 status pre-operatively as an auxiliary diagnosis to CNB assessment.

**Methods:**

Among 4211 breast cancer patients cataloged in the Nation-wide Multicenter 10-year Retrospective Clinical Epidemiological Study of Breast Cancer in China, 2291 patients with complete relevant information were included in this study, which were further randomized 3:1 and divided into a training set and a validation set. The nomogram was established based on independent predictors of HER2 positivity recognized by logistic regression analysis and further validated internally and externally.

**Results:**

The multivariate logistic regression analysis showed that T-stage, N-stage, estrogen receptor (ER) status, progesterone receptor (PR) status were independent predictors for HER2 status. The nomogram was thereby constructed by those independent predictors as well as histology type. The areas under the receiver operating characteristic curve (AUC) of the training set and the validation set were 0.636 and 0.681, respectively. The calibration plots demonstrated good fitness of the nomogram for HER2 status prediction. With the optimal cutoff value, the nomogram yielded 80.0% sensitivity, 43.1% specificity in the training set and 81.1% sensitivity, 49.8% specificity in the validation set.

**Conclusions:**

The present nomogram can provide valuable information on HER2 status and combined with standard CNB assessment, clinicians could make more appropriate decision on neoadjuvant therapy of breast cancer.

**Electronic supplementary material:**

The online version of this article (10.1186/s13000-019-0806-4) contains supplementary material, which is available to authorized users.

## Background

Breast cancer has been reported to be the most common malignant tumor and the leading cause of cancer death among women worldwide [1]. The utilization of neoadjuvant therapy (preoperative systemic therapy) provide the opportunity to monitor response during treatment, and help to increase the curative intervention as well as the breast conservation rate by reducing tumor burden [2]. The roles of CNB have been well established as an important preoperative diagnostic method for breast lesions. CNB is less invasive than excision biopsy and provide more information than fine needle aspiration (FNA). In the case of neoadjuvant therapy of breast cancer, CNB is the gold standard for pathological diagnosis and molecular subtype assessment.

Emerging data show that neoadjuvant chemotherapy combined with HER2-targeted therapy yields a higher pathologic complete response (pCR) rate and favorable disease-free and overall survival when compared with neoadjuvant chemotherapy alone in women with HER2-positive breast cancers [3–8]. The increasing number of HER2 positive breast cancer patients being treated in the neoadjuvant setting gives rise to the need to accurately assess HER2 status on the CNB material that often is the only tissue available before treatment in these patients. However, due to intratumoral heterogeneity, the concordance rate of HER2 status between CNB and subsequent excisional biopsies of the same tumor varies from 81 to 96% as reported by different institutions [9–12].

In order to improve the accuracy of HER2 assessment in CNB specimens so that patients could get appropriate neoadjuvant therapy, we intend to identify possible predictors based on a nation-wide multicenter data spans 10 years in China and construct a nomogram for predicting HER2 status, which will increase the accuracy of HER2 assessment pre-operatively. In combination with CNB and nomogram prediction, it may provide more accurate information on whether the HER2 targeted therapy is needed in the neoadjuvant setting.

## Methods

### Study design and data collection

Data was obtained from the hospital-based, multicenter, 10-year (1999–2008), retrospective study of randomly selected pathology confirmed primary female breast cancer cases via medical chart review which was approved by the ethics committee of the Cancer Foundation of China. In order to obtain the population, China was stratified into 7 geographic regions according to the traditional administrative district definition (north, northeast, northwest, middle, east, south and southwest); these regions extend over the majority of the country and represent different levels of breast cancer burden (Additional file 1: Figure S1) [13]. Of these patients, 4211 patients with a median age of 48 years (range: 21–86 years) were enrolled in this study. The age distribution of these patients conformed to 1999–2008 population-based breast cancer incidence data retrieved from the National Central Cancer Registry database (Additional file 1: Figure S2) [14]. One university hospital with good standard quality from each region was selected to provide the required study cases. Hospital records were reviewed by local clerks within each hospital according to the designated protocol. Further methodological details including patients selection, pathologic diagnostic criteria, data collection and quality control can be found in our previously published papers [13, 15].

Among overall population of 4211 patients, 2291 patients with complete relevant information (age, BMI, location of lesions, local infiltration, T-stage, N-stage, ER, PR, HER2 and histologic type) were included in this study, which were further randomized 3:1 and divided into a training set (*N* = 1718) and a validation set (*N* = 573).

In this study, IHC method was used to determine the HER2 status, scores of 0 and 1+ be regarded as HER2-negative and that HER2 scores of 3+ be considered as HER2-positive. HER2 score of 2+ is regarded as HER2-borderline and HER2-borderline cases were excluded in our cohort due to low utilization rate of FISH assay during 1999–2008 in China.

### Nomogram construction and validation

To develop a well-calibrated nomogram for predicting the probability of HER2 positivity, univariate as well as multivariate logistic regression analyses were performed to screen the predictors for HER2 positivity. Independent predictors (*P* < 0.05 in the multivariate logistic regression analysis) as well as clinical significant predictors were included in the nomogram construction. The Hosmer and Lemeshow test was applied to assess goodness of fit of the model, and *P* > 0.05 indicated a good fit. The odds ratios (ORs) and 95% confidence intervals (CIs) were also calculated.

The nomogram was validated internally in the training set and externally in the validation set. A receiver-operating characteristic (ROC) curve was used to evaluate the effectiveness of the nomogram. Area under the curve (AUC) was calculated. The AUC ranged from 0 to 1, with 1 indicating perfect concordance, 0.5 indicating no better than chance, and 0 indicating discordance. Statistical differences between different AUCs were investigated by the DeLong method. The calibration plot with bootstrapping was used to illustrate the association between the actual probability and the predicted probability.

All reported *P* values are two-sided. The statistical analysis was carried out using SPSS (version 19.0; SPSS Company, Chicago, IL) and R software version 3.4 (http://www.r-project.org).

## Results

### Clinicopathological features

**Fig. 1:**
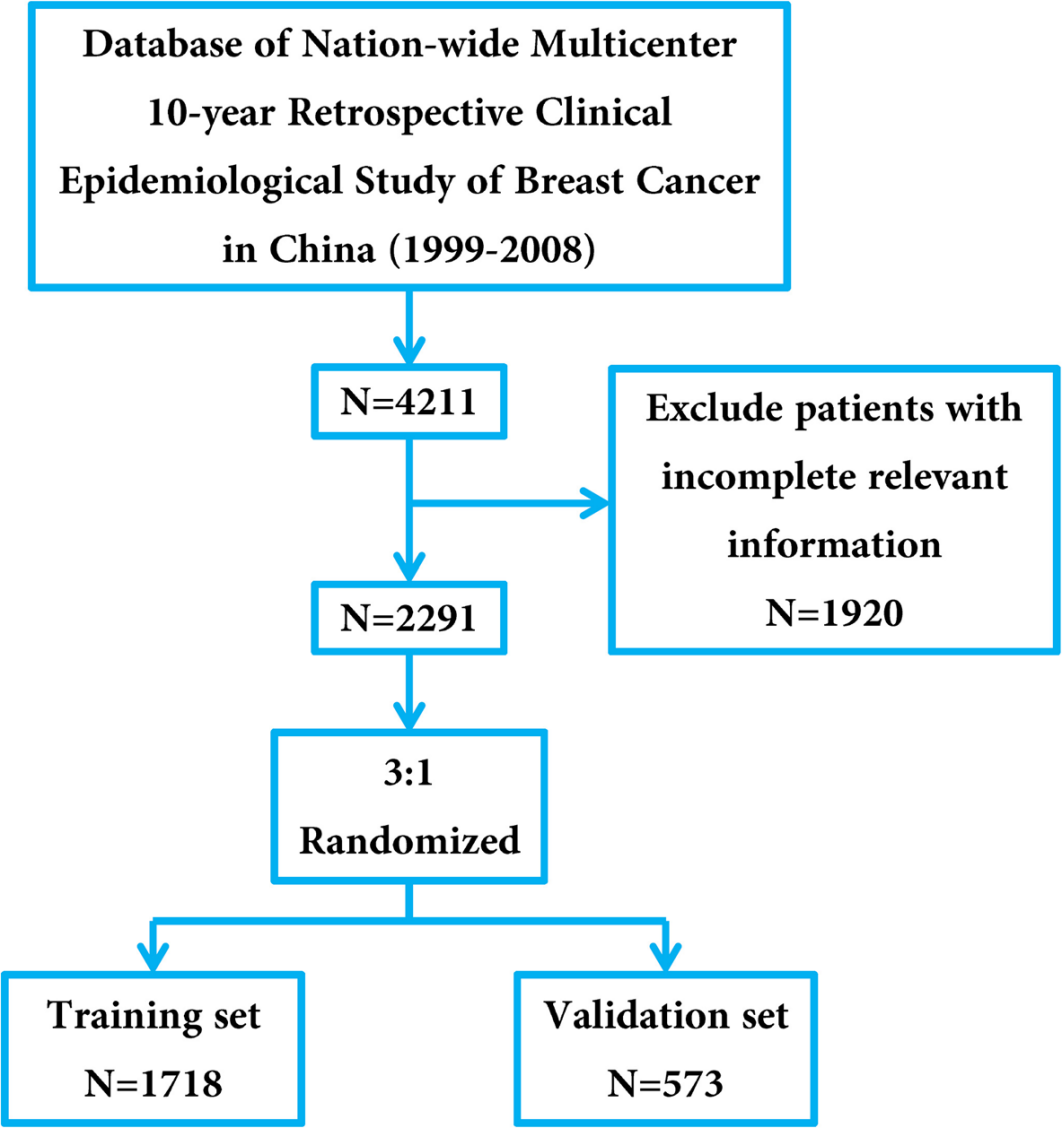
Flow diagram of the study design. A total of 2291 patients with complete relevant information were enrolled in this study and were randomized 3:1 and divided into a training set (*N* = 1718) and a validation set (*N* = 573)

There were 4211 breast cancer patients cataloged in the Nation-wide Multicenter 10-year Retrospective Clinical Epidemiological Study of Breast Cancer in China. After excluding 1920 patients with incomplete relevant information, 2291 eligible patients were included in the study which were randomized 3:1 and divided into a training set (*N* = 1718) and a validation set (*N* = 573) (Fig. 1). The clinicopathological features were similar between the training set and the validation set (Table 1).

**Table 1:** Clinicopathological characteristics of the study populations

	Training set		Validation set		*P*
	N	mean ± SD	N	mean ± SD	
		%		%	
Age	1718	49.81 ± 10.416	573	48.96 ± 10.386	0.090
BMI	1540	23.47 ± 3.184	508	23.32 ± 3.074	0.358
Location of Lesions					0.781
UOQ	814	47.38	272	47.47	
UIQ	298	17.35	90	15.71	
LIQ	100	5.82	32	5.58	
LOQ	180	10.48	64	11.17	
Central	220	12.81	71	12.39	
N/A	106	6.17	44	7.68	
T-Stage^1^					0.684
T1	535	31.14	183	31.94	
T2	986	57.39	333	58.12	
T3	131	7.63	35	6.11	
T4	66	3.84	22	3.84	
Local Infiltration					0.685
Skin & Chest Wall	4	0.23	1	0.17	
Only Skin	53	3.08	20	3.49	
Only Chest Wall	9	0.52	1	0.17	
No	1652	96.16	551	96.16	
N-Stage^a^					0.031
N0	1122	65.31	412	71.90	
N1	399	23.22	110	19.20	
N2	133	7.74	32	5.58	
N3	64	3.73	19	3.32	
Histology					0.161
CIS-Mi	48	2.79	19	3.32	
IDC	1503	87.49	493	86.04	
ILC	50	2.91	27	4.71	
Others^b^	117	6.81	34	5.93	
ER					0.648
Positive	1020	59.37	334	58.29	
Negative	698	40.63	239	41.71	
PR					0.704
Positive	1037	60.36	351	61.26	
Negative	681	39.64	222	38.74	
HER2					0.122
Positive	436	25.38	127	22.16	
Negative	1282	74.62	446	77.84	

^a^T-stage and N-stage were both clinical stage determined by clinical (physical examination or radiologic) measurements

^b^Others: tubular carcinoma, mucinous carcinoma, medullary carcinoma

Abbreviations: *BMI* body mass index, *LIQ* lower-inner quadrant, *LOQ* lower-outer quadrant, *UIQ* upper-inner quadrant, *UOQ* upper-outer quadrant, *N/A* not available, *CIS-Mi* ductal/lobular carcinoma in situ and microinvasive carcinoma; *IDC* infiltrating ductal carcinoma, *ILC* infiltrating lobular carcinoma, *ER* estrogen receptor, *PR* progesterone receptor, *HER2* human epidermal growth factor receptor-2

### Predictors for HER2 positive breast Cancer

We used logistic regression as univariate and multivariate analysis to evaluate the risk factors for HER2 positive breast cancers in the training set. T-stage, N-stage, ER and PR were risk factors for HER2 positive breast cancer in the univariate analysis, and these variables were further confirmed as independent risk factors in multivariate analysis (Table 2). According to the results, ER positive patients (*P* = 0.01, OR = 0.690 [95% CI: 0.522–0.914]) and PR positive patients (*P* = 0.025, OR = 0.726 [95% CI: 0.548–0.961]) were less likely to be HER2 positive than ER negative patients and PR negative patients, and patients with T2 (*P* = 0.009, OR = 0.720 [95% CI: 0.562–0.921]), T3 (*P* = 0.045, OR = 0.623 [95% CI: 0.392–0.989]) and T4 (*P* = 0.034, OR = 0.493 [95% CI: 0.256–0.949]) stage were less likely to be HER2 positive compared with those with T1 stage. In contrast, patients of N2 (*P* = 0.005, OR = 1.747 [95% CI: 1.182–2.583]) and N3 (*P* = 0.000, OR = 2.866 [95% CI: 1.683–4.879]) stage were more likely to have HER2 positive breast cancer than patients of N0 stage. Histology was not significantly associated with HER2 positivity.

**Table 2:** Analysis of risk factors for HER-2 positivity

	Univariate analysis		Multivariate analysis	
	OR (95% CI)	*P*	OR (95% CI)	*P*
Age	0.994 (0.984–1.005)	0.297		
BMI	0.995 (0.960–1.031)	0.769		
Location of Lesions				
UOQ	1			
UIQ	1.094 (0.813–1.471)	0.554		
LIQ	1.030 (0.645–1.646)	0.900		
LOQ	0.822 (0.561–1.204)	0.313		
Central	0.600 (0.411–0.877)	0.008		
N/A	1.100 (0.701–1.725)	0.679		
T-Stage				
T1	1		1	
T2	0.787 (0.621–0.998)	0.048	0.720 (0.562–0.921)	0.009
T3	0.767 (0.492–1.196)	0.242	0.623 (0.392–0.989)	0.045
T4	0.607 (0.322–1.145)	0.123	0.493 (0.256–0.949)	0.034
Local Infiltration				
No	1			
Only Skin	0.761 (0.388–1.492)	0.426		
Only Chest Wall	0.830 (0.172–4.012)	0.817		
Skin & Chest Wall	0.000	0.999		
N-Stage				
N0	1		1	
N1	1.165 (0.895–1.517)	0.256	1.172 (0.892–1.540)	0.255
N2	1.830 (1.249–2.681)	0.002	1.747 (1.182–2.583)	0.005
N3	2.605 (1.559–4.351)	0.000	2.866 (1.683–4.879)	0.000
Histology				
CIS-Mi	1		1	
IDC	0.990(0.518–1.890)	0.975	0.919(0.475–1.777)	0.801
ILC	0.367(0.127–1.064)	0.065	0.379(0.129–1.110)	0.077
Others	0.337(0.143–0.794)	0.013	0.296(0.124–0.710)	0.006
ER				
Negative	1		1	
Positive	0.591(0.475–0.736)	0.000	0.690(0.522–0.914)	0.010
PR				
Negative	1		1	
Positive	0.602(0.483–0.750)	0.000	0.726(0.548–0.961)	0.025

Logistic regression analysis was used for univariate and multivariate analysis of different variables predicting HER2 positivity

### Construction and validation of the nomogram

**Fig. 2:**
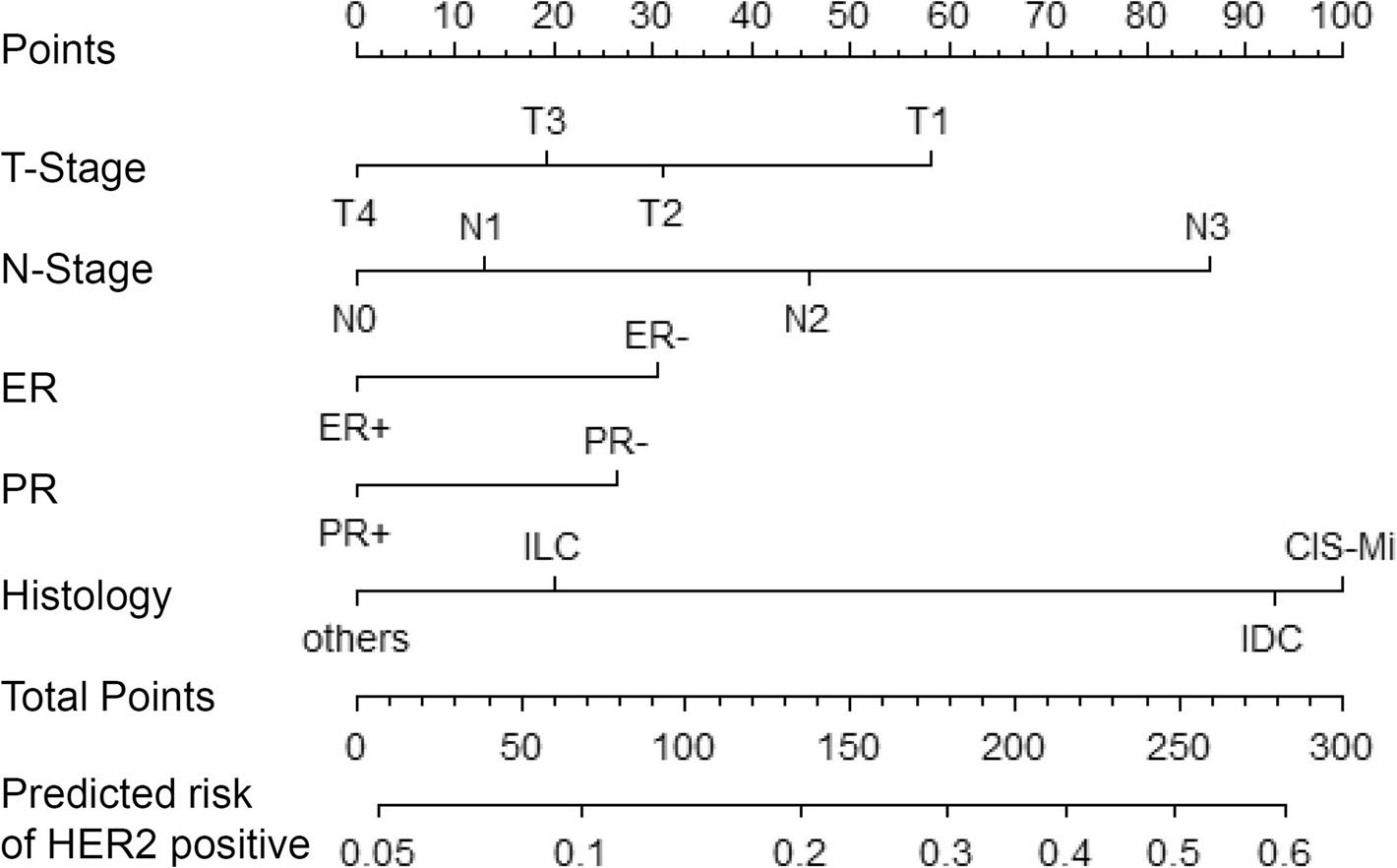
A nomogram to predict the probability of HER2 positive breast cancer

Based on the results of the multivariate analysis, a nomogram was constructed with independent predictors for HER2 status including T-stage, N-stage, ER status and PR status as well as histology (Fig. 2). A total points is the sum of points for each variable (top plotting scale) and the probability of HER2 positivity is the corresponding number of the total points in the nomogram (bottom plotting scale). The *P*-value of the Hosmer and Lemeshow test was 0.055, indicting a good statistical fit.

**Fig. 3:**
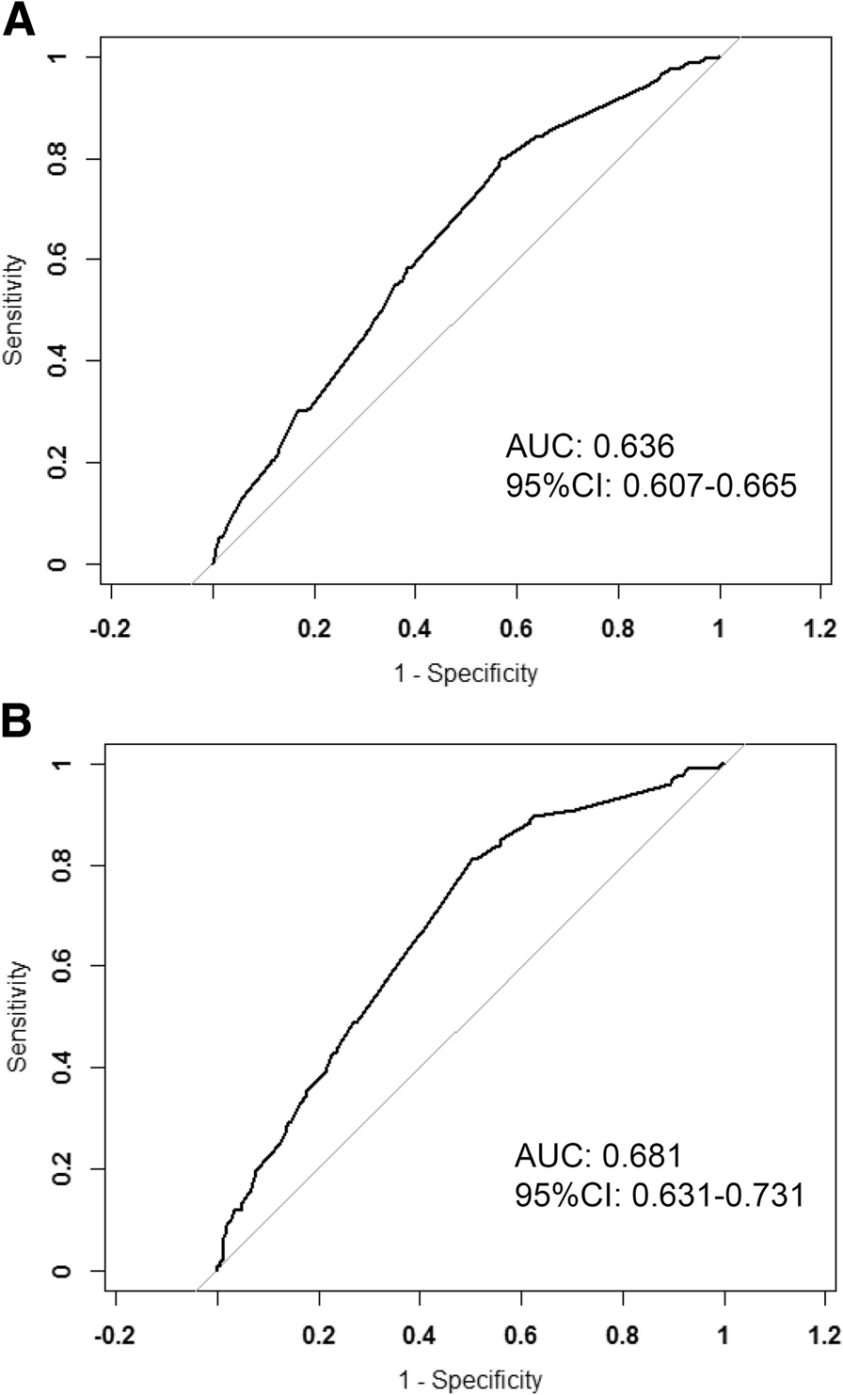
Validation of the nomogram. (**a**) Internal validation using the ROC curve in the training set. The area under the ROC curve (AUC) is 0.636, 95% confidence interval (95% CI, 0.607–0.665). (**b**) External validation using ROC in the validation set. The AUC is 0.681 (95% CI, 0.631–0.731)

**Fig. 4:**
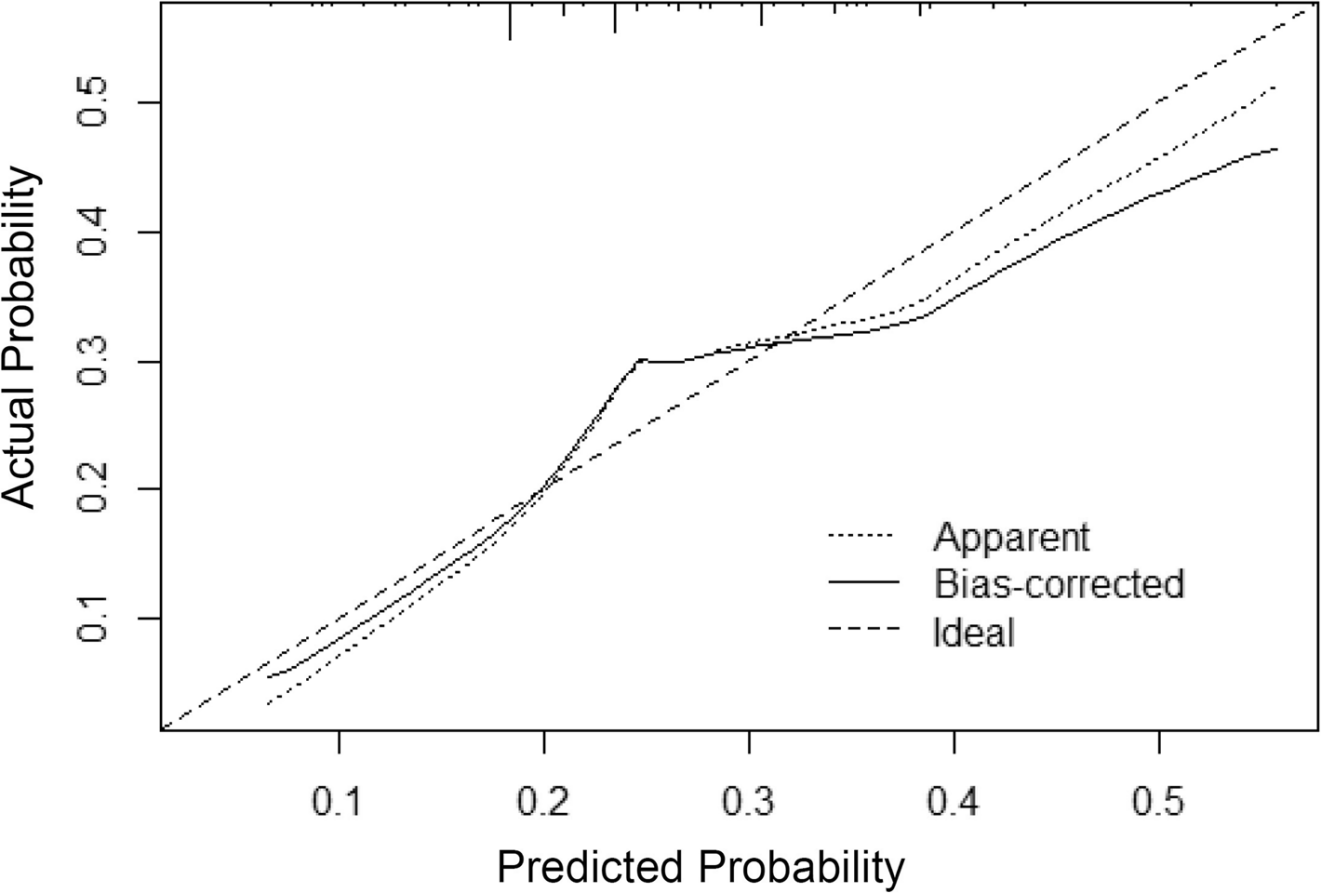
Calibration plots of the nomogram for the probability of HER2 positive breast cancer (bootstrap 1000 repetitions)

The ROC curve analysis was performed to validate the nomogram internally in the training set (Fig. 3a) and externally in the validation set (Fig. 3b). In the training set, the AUC was 0.636 (95% CI: 0.607–0.665). In the validation set, the AUC was 0.681 (95% CI: 0.631–0.731). There’s no statistical difference between two AUCs (*P* = 1). The calibration plot (Fig. 4) indicated that the nomogram was well calibrated.

As shown in Table 3, higher cutoff value resulted in the increasing of specificity and positive predictive value, while sensitivity and negative predictive value decreased. According to the Youden’s method [16], optimal cutoff values of the training and validation sets were 0.212 (sensitivity: 80.0%, specificity: 43.1%, positive predictive value: 32.4%, negative predictive value: 86.4%) and 0.204 (sensitivity: 81.1%, specificity: 49.8%, positive predictive value: 31.5%, negative predictive value: 90.2%), respectively (Table 4).

**Table 3:** Values of sensitivity, specificity, and predictive values of the predicted probability at different cutoff values

Predicted probability	Training set				Validation set			
	sensitivity	specificity	PPV	NPV	sensitivity	specificity	PPV	NPV
≥0.1	98.9%	4.8%	26.1%	92.5%	98.4%	4.5%	22.7%	90.9%
≥0.2	83.9%	36.5%	31.0%	87.0%	83.5%	34.3%	26.6%	87.9%
≥0.3	44.0%	70.8%	33.9%	78.8%	43.3%	71.7%	30.4%	81.6%
≥0.4	9.6%	95.9%	44.2%	75.7%	10.2%	97.5%	54.2%	79.2%
≥0.5	4.8%	98.7%	53.8%	75.3%	4.7%	99.5%	66.7%	78.5%

*NPV* negative predictive value, *PPV* positive predictive value

**Table 4:** Values of sensitivity, specificity, and predictive values of the predicted probability at the optimal cutoff value

	The Optimal Cutoff	Sensitivity	Specificity	PPV	NPV
Training set	0.212	80.0%	43.1%	32.4%	86.4%
Validation set	0.204	81.1%	49.8%	31.5%	90.2%

*NPV* negative predictive value, *PPV* positive predictive value

*The optimal cutoff value is determined according to the Youden’s method

### The application of the nomogram

To display the application of the nomogram, we took ten patients who were tested for HER2 status in tissues from both the core needle biopsy and the excision as examples (Table 5). Patients 1–4 have concordant HER2 status in CNB specimens, excisional specimens and nomogram prediction. Patients 5 and 6 were HER2 positive in the CNB specimens, but HER2 negative in the excisional specimens which were concordant with our prediction done by the nomogram. Patient 7 was HER2 negative in the CNB specimen, but HER2 positive in the excisional specimen which was concordant with our prediction done by the nomogram. Patients 8–10 were HER2-borderline in the CNB specimens, by using the nomogram, prediction on HER2 status were consistent with the result in excisional specimens.

**Table 5:** The application of the nomogram

	Points					Patients					
		1	2	3	4	5	6	7	8	9	10
T-Stage											
T1	58		√						√	√	
T2	30				√		√				√
T3	20			√		√		√			
T4	0	√									
N-Stage											
N0	0			√			√		√	√	√
N1	12.5				√	√					
N2	46	√	√					√			
N3	87										
ER											
+	0		√		√	√	√	√	√		√
-	30	√		√						√	
PR											
+	0		√		√	√	√	√	√		√
-	27.5	√		√						√	
Histology											
CIS-Mi 100							√				
IDC	94	√	√	√	√	√		√		√	
ILC	20								√		√
Others 0											
Total points		197.5	198	171.5	136.5	126.5	130	160	78	209.5	50
Predictive probability of HER2 positive											
High		√	√	√				√		√	
Low					√	√	√		√		√
HER2 status in CNB tissue											
+		√	√	√		√	√				
+/−									√	√	√
-					√			√			
HER2 status in excision tissue											
+		√	√	√				√		√	
-					√	√	√		√		√

## Discussion

In our current research, we first randomized the population containing 2291 patients with completed relevant information into a training set and a validation set. Based on the multivariate logistic regression analysis, we identified independent variables for predicting the HER2 status. Patients who were ER positive, PR positive were less likely to be HER2 positive than those who were ER negative, PR negative. Patients who were of T1 stage were more likely to be HER2 positive than those who were of T2, T3 and T4 stage. Patients with N2, N3 stage were more likely to be HER2 positive than those with N1 stage. Histology type did not show statistically significance in the multivariate logistic regression analysis. Next, we constructed a nomogram on the basis of these predictors as well as clinical significant predictors (histology). The AUCs in the training (internal validation) and validation sets (external validation) were 0.636 and 0.681, respectively.

Tissue diagnosis of breast cancer by CNB before proceeding to any kind of treatment is the gold standard of modern medical practice according to clinical practice guidelines. The accuracy of histologic identification by CNB is superior to the cytological diagnosis by fine needle aspiration and CNB specimens could provide more tissue for further immunohistochemical (IHC) assessment if neoadjuvant therapy is indicated. Accordingly, physicians can discuss the treatment plan with the patient preoperatively and give her the chance of one-step operation or perform the neoadjuvant treatment based on the assessment of ER, PR, HER2 and Ki67 status in the CNB samples. In cases of pCR, this will be the only source for the evaluation of breast cancer molecular subtype. It can also determine the molecular subtype more accurately than the IHC exam of an excised tumor with partial response to neoadjuvant chemotherapy.

Data from clinical trials have shown that patients with HER2 positive breast carcinomas have significantly better responses (more frequently obtaining pathologic complete response and greater percent disease-free survival) when treated with HER2 targeted therapy simultaneously with neoadjuvant chemotherapy than with neoadjuvant chemotherapy alone [17]. With the increasing use of neoadjuvant therapies, clinicians require accurate information on HER2 status at the time of CNB as false negative HER2 result might impair the potential benefit from HER2 targeted therapy for patients. Concordance rate of HER2 status between CNB and subsequent excisional biopsies of the same tumor varies from 81 to 96% as reported by different institutions [9–12]. The disconcordance between CNB and excisional biopsy were mainly due to intratumoral heterogeneity. Studies showed that to improve the accuracy of HER2 status as well as other important information from CNB, a minimum of four cores is required [18]. And when comparing 14-, 16-, 18-gauge needles, the accuracy rose with needles of increasing size [19]. These results suggested that diagnostic accuracy of CNB increased with the increase of harvested specimens.

However, improving the accuracy of HER2 status in CNB specimens by increasing the cores and needle size is invasive and may cause more complications after CNB, so we developed noninvasive method - a nomogram to predict the HER2 status in the entire tumor prior to the surgery. If the HER2 status from CNB specimens is different from the nomogram predicted, then additional CNB may be needed to verify the exact status of HER2, in order to avoid inappropriate neoadjuvant therapy caused by false negative assessment of HER2.

In our study, we analyzed the common clinicopathological features including age, BMI, location of tumor, T-stage, local infiltration, N-stage, histology type, ER expression and PR expression in HER2-positive and -negative cohort, and found that ER negative, PR negative, T1 stage and N2/N3 stage were independent risk factors for HER2 positive breast cancer. Traina et al. demonstrated that HER2 overexpression was significantly correlated with negative hormone receptor (HR) status, positive nodal status and G3 tumor grade based on data from 1355 Italian breast cancer patients [20]. Study from Morocco which included 1508 patients found that overexpression of HER2 was associated with high tumor grade, vascular space invasion and ER negativity significantly [21]. Another study from a single center in China suggested that ER status, PR status and tumor grade were significantly associated with HER2 status [22]. Different studies from different institution indicated the same finding that HER2 overexpression is correlated with HR negativity, while the relationship between HER2 expression and other clinicopathologic parameters varies, such as tumor dimension, nodal involvement and tumor grading.

The nomogram, a simple graphical prediction tool, allows oncologists to assess the predictive risk of individuals [23]. And it’s been considered as an important component of modern medical decision making [24]. Another advantage of the nomogram is that it is noninvasive. So far, this is the first nomogram constructed to predict HER2 status based on nation-wide multicenter data in breast cancer. The nomogram we constructed here is not aimed to replace the molecular test of HER2, but to prevent the false negative of HER2 due to CNB sample limitation. According to the Youden’s method [16], the optimal cutoff value of the training and validation set in this study were 0.212 and 0.204, at which the sensitivity were 80 and 81.1% while the specificity were only 43.1 and 49.8%, respectively. As we can’t have both high sensitivity and high specificity, higher sensitivity is what we more needed considering that the purpose of our nomogram is to prevent false negativity. By using the optimal cutoff value and the nomogram, we predicted HER2 status correctly in several patients of different TNM stage and HR status. Since there’re many rural areas in China that do not have sufficient resources and medical insurance coverage, our prediction model has practical value. For patients who cannot afford the FISH assay and trastuzumab therapy, our model will be helpful for predicting their HER2 status. If a patient with IHC-determined HER2-borderline disease were predicted to be HER2-positive and that patient could not afford trastuzumab, a stronger chemotherapy regimen, e.g., dose-dense AC-T, could be considered as an alternative to TC regimen.

One major limitation of our study was that we couldn’t analyze other important pathological parameters such as tumor grade and Ki67 due to incomplete information. The database consisted of breast cancer patients diagnosed during 1999 to 2008 when the pathologic diagnosis of breast cancer in China was rapidly developing and the parameters may vary in different institution at that time, so the tumor grade, Ki67 and other parameters which are proved of prognostic importance were not included in this retrospective study. Further effort is required to improving the database by adding new cases with complete information, so that we may find more risk factors for HER2 positive breast cancer and the nomogram may be adjusted to be more accurate, hence, patients could obtain more benefit from this nomogram by given the right therapy regimens.

## Conclusions

The disconcordance of HER2 status between CNB and excisional biopsies of the same tumor may cause inappropriate neoadjuvant therapy in women with HER-2 positive breast cancer. Based on the nation-wide multicenter data spans 10 years in China, we found that ER negative, PR negative, T1 stage and N2/N3 stage were independent risk factors for HER2 positive breast cancer. We further establish the nomogram for HER2 status prediction which is validated both internally and externally. The nomogram could be a valuable tool for improving the accuracy of HER2 assessment pre-operatively. By combining CNB and the nomogram, clinicians could get more information of one individual patient and provide more suitable treatment accordingly.

## Additional file

Additional file 1: **Figure S1.** Geographic distribution of sites included in the study ^1^. **Figure S2.** The age distribution of breast cancer in Chinese women^2^. (DOCX 459 kb)

## Abbreviations

AUC: The areas under the receiver operating characteristic curve
CIs: Confidence intervals
CNB: Core needle biopsy
ER: Estrogen receptor
FNA: Fine needle aspiration
HER2: Human epidermal growth factor receptor 2
HR: Hormone receptor
IDC: Invasive ductal carcinoma
IHC: Immunohistochemical
ORs: Odds ratios
pCR: Pathologic complete response
PR: Progesterone receptor
ROC: Receiver-operating characteristic
