# Supplementary material for: A nomogram for predicting the HER2 status in female patients with breast cancer in China: a nationwide, multicenter, 10-year epidemiological study
Source: Diagn Pathol. 2019 May 4;14:35. doi: 10.1186/s13000-019-0806-4 (PMC6500005; doi:10.1186/s13000-019-0806-4)
Supplement: Supplementary file 1 — Figure S1. Geographic distribution of sites included in the study 1. Figure S2. The age distribution of breast cancer in Chinese women2. (DOCX 459 kb) [file 13000_2019_806_MOESM1_ESM.docx]

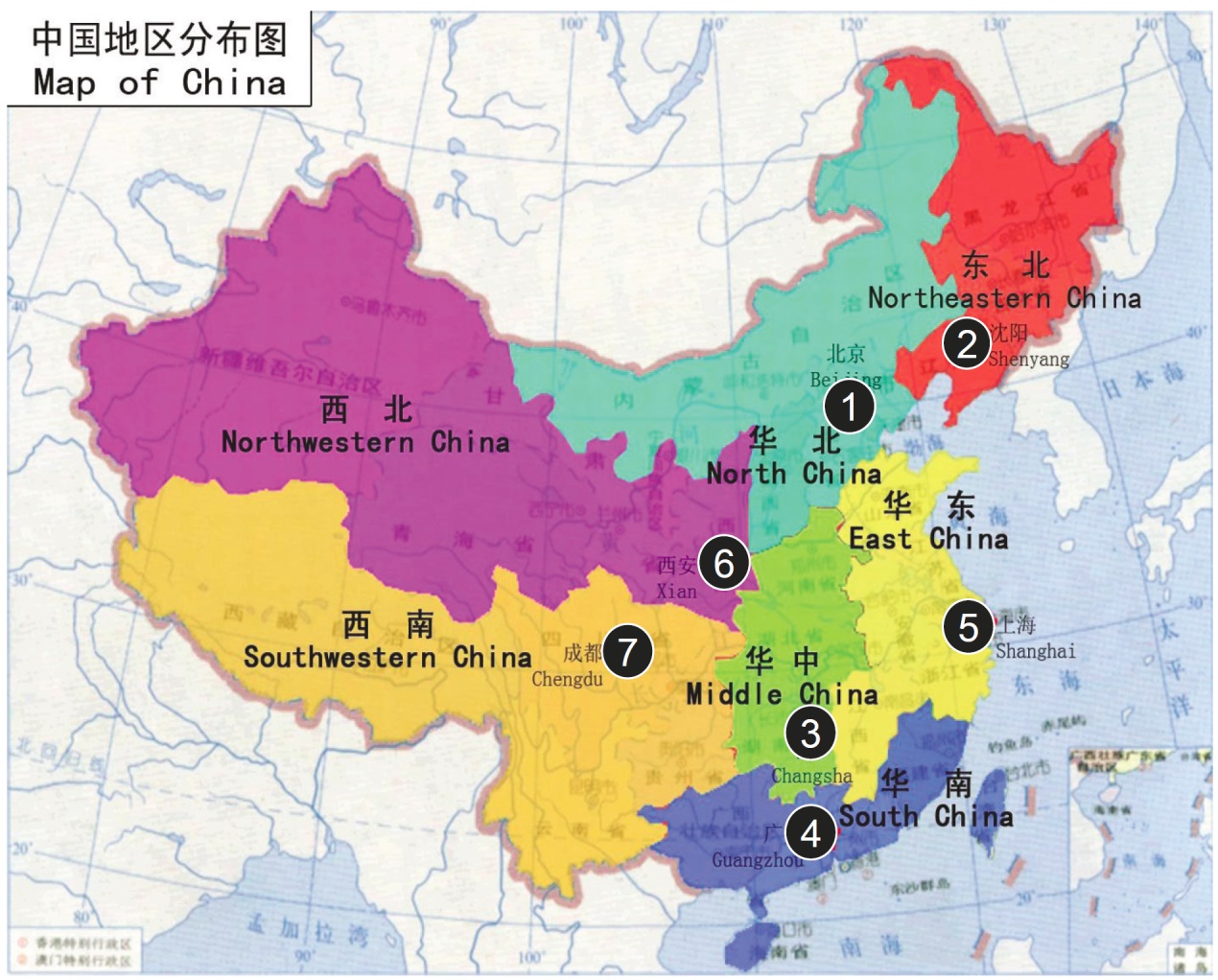


Figure S1. Geographic distribution of sites included in the study. 1: Cancer Institute/ Hospital, Chinese Academy of Medical Sciences 2: Liaoning Cancer Hospital 3: Second Xiangya Hospital, Central South University 4: Guangdong Sun Yat-Sen University Cancer Center 5: Zhejiang Cancer Hospital 6: First Affiliated Hospital of Xi’an Jiaotong University 7: Sichuan Cancer Hospital[^1^](#_ENREF_1).


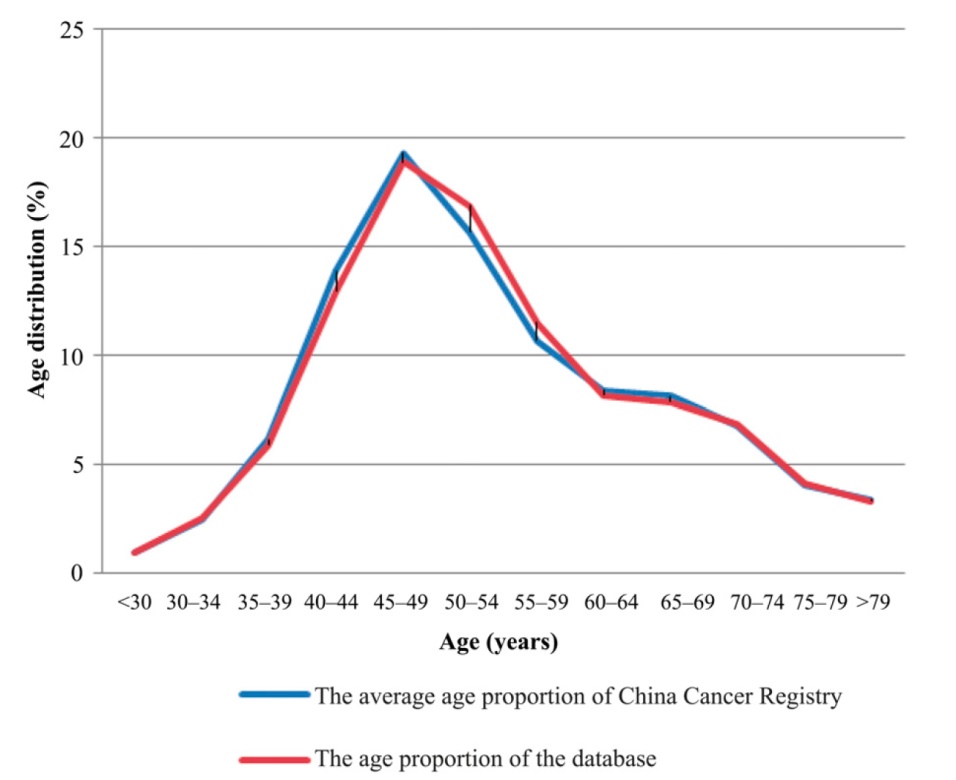


Figure S2. The age distribution of breast cancer in Chinese women[^2^](#_ENREF_2).

References:

1 Li J, Zhang B-N, Fan J-H, Pang Y, Zhang P, Wang S-L *et al*. A Nation-Wide multicenter 10-year (1999-2008) retrospective clinical epidemiological study of female breast cancer in china. BMC Cancer 2011; 11: 364.

2 Zhang B-L, Sivasubramaniam PG, Zhang Q, Wang J, Zhang B, Gao J-D *et al*. Trends in Radical Surgical Treatment Methods for Breast Malignancies in China: A Multicenter 10-Year Retrospective Study. The Oncologist 2015; 20: 1036-1043.
